# Organic Eluates Derived from Intermediate Restorative Dental Materials

**DOI:** 10.3390/molecules25071593

**Published:** 2020-03-30

**Authors:** Triantafyllia Vouzara, Konstantina Roussou, Alexandros K. Nikolaidis, Kosmas Tolidis, Elisabeth A. Koulaouzidou

**Affiliations:** 1 Division of Dental Tissues’ Pathology and Therapeutics (Basic Dental Sciences, Endodontology and Operative Dentistry), School of Dentistry, Aristotle University of Thessaloniki, 541 24 Thessaloniki, Greece; fivou@yahoo.gr (T.V.); roussaki90@gmail.com (K.R.); nikolchem@dent.auth.gr (A.K.N.); ktolidis@dent.auth.gr (K.T.); 2 Department of Pediatric Dentistry, School of Dentistry, Aristotle University of Thessaloniki, Thessaloniki 541 24, Greece

**Keywords:** gas chromatography, organic eluates, intermediate restorative dental materials

## Abstract

A great number of different types of materials have been used in dentistry as intermediate restoratives. Among them, new resin-based bases have been released in the dental market. The present study focuses on the identification of the organic eluates released from such materials and the study of their surface microstructure in combination with their corresponding elemental composition. For this purpose, the following materials were used:ACTIVA™BioACTIVE-BASE/LINER™, Ketac™Bond Glass Ionomer, SDR™ and Vitrebond™Light Cure Glass Ionomer Liner/Base. Methanolic leachates derived from polymerized materials were analyzed by means of gas chromatography-mass spectrometry (GC-MS). Scanning electron microscopy(SEM) was used for the surface monitoring of suitably prepared specimens. The GC-MS analysis revealed the elution of twenty different substances from the three resin-based materials, while none was eluted from the glass ionomer base. The SEM analysis for Vitrebond™ presented small pits, the one for Ketac™Bond presented elongated cracks, while no voids were present for ACTIVA™BioACTIVE-BASE/LINER™ and SDR™. Moreover, the resin matrix of some dental materials may inhibit elements’ accumulation on the surface layers. Particularly, the detected organic eluents may be related to potential toxic effects.

## 1. Introduction

In restorative dentistry a wide range of materials, called intermediate restoratives, are placed upon the dentine prior to the placement of the final restoration. In this context, two main treatments may be applied in clinical practice: (a) direct pulp capping, where the material’s placement is in direct contact with the pulp tissue, and (b) indirect pulp capping, where there is a remaining dentin layer between the pulp and the material.Both procedures aim to protect the dental pulp and preserve vitality [1,2]. These intermediate restoratives are often referred to as cavity liners or bases. Calcium hydroxide is the “gold standard” material, since it is traditionally used and has exhibited clinical success [3]. However, in the last decades new materials consisting mainly of calcium silicates, such as mineral trioxide aggregate (MTA), have been introduced in the clinical practice and have exhibited high success rates [4,5,6]. MTA and calcium hydroxide induce the formation of hydroxyapatite, release calcium ions and promote dentin bridge formation with less inflammatory response [1]. Additionally, both of these materials are proved to have a minimum cytotoxic action to the dental pulp tissue [7]. However, both MTA and calcium hydroxide have some disadvantages, like long-setting times or alack of setting, a high solubility, a gradual resorption, weak physical properties and poor handling [2]. 

In order to overcome the previously mentioned disadvantages, several other types of materials are available in the market [1]. Glass ionomer cements (GICs), resin-modified glass ionomer cements (RMGICs) and flowable composites have been proposed for such applications. GICs are composed of a degradable glass and a polymeric acid, and an acid base reaction phase during their setting proceduretakes place [8,9]. The benefits of the GICs’ useare the chemical bond to the tooth structure, the fluoride ions release, which causes bioactivity and caries protection, and their physical properties which are similar to those of dentin [10,11,12,13]. The disadvantages of GICs are their poor aesthetic and mechanical features, something that led to the development of RMGICs [14,15]. 

RMGICs are hybrid materials that consist of the GICs’ components enriched with resin monomers and initiators [9,16]. Resin-modified materials can be immediately light cured, which provides better handling and greater accuracy in placement [17]. Although these materials present superior physical and mechanical properties [17], they are considered to exhibit some cytotoxic action and are associated with rather negative clinical outcomes [6,18,19]. The cytotoxic action of these resin-based materials is attributed to the monomers and organic eluates that may be released [20].

During the last years, bulk-fill resin-based composites (RBCs) have emerged in clinical practice due to their simplicity. They contain different modified proprietary resins, modulators and fillers with an increased size and decreased load. Manufacturers allege that these formulations might enhance the depth of cure by up to 4 mm [21,22]. However, there are concerns about the complete polymerization and the presence of unreacted monomers in the mass of these materials [23,24].

Recently, bioactive glass has been added into dental materials, and it has been stated that these materials have the capacity to promote hard tissue formation and mineralization [25]. The first series of restorative products that, according to manufacturers, contain bioactive glass fillers has been released in the dental market under the trade name Activa^TM^ Bioactive (Pulpdent, Watertown, MA, USA). They contain patented bioactive ionic and moisture-friendly resin, patented rubberized resin and reactive glass fillers, while they are free of bisphenol A glycol dimethacrylate (Bis-GMA), bisphenolA (BPA) and its derivatives. Their setting process is a combination of three chemical reactions: acid-base reaction, light-polymerization and chemical curing [26,27]. They take part in the pH cycles of ionic exchange between teeth and saliva, as they release and recharge with calcium, phosphate and fluoride ions. They are promoted to be bioactive and to form mineral apatite in contact with the tooth, a procedure that enhances the sealing ability of the materials. The company claims that its products blend the advantages of composites (strength, esthetics and physical properties) with the benefits of GICs (fluoride release). Activa-BioACTIVE BASE/LINER^TM^ is intended to be used instead of glass ionomers, resin-modified glass ionomers and flowable composites, without etching or bonding agents. According to the Material Safety Data Sheet (MSDS), Activa-BioACTIVE BASE/LINER^TM^ is a resin-based material that contains diurethane and other methacrylates with modified polyacrylic acid. In addition, this product comprises silica and fluoride ions, which could behave as unpolymerized monomers after the polymerization process. Taking into account the composition of this material, its chemical propertiesand the possible interactions with media, simulating the oral environment should be researched in comparison with other materials used for the same applications. 

In order to identify the eluates deriving from resin-based materials, different analytical methods, such as high performanceliquid chromatography (HPLC) and gas chromatography (GC) are proposed [28]. The selection of the proper analytical method is partly based on the molecular size of the organic components in question. For example, large molecular size compounds, such as Bis-GMA, urethane dimethacrylate (UDMA) and bisphenol A ethoxylated dimethacrylate (Bis-EMA), are preferably detected by the use of HPLC, while smaller and volatile compounds are preferably detected by the use of GC [29]. 

Monomers released from the dental resin matrix may influence cells’ viability and other biological functions [30]. Two of the most commonly released monomers are 2-hydroxyethyl methacrylate (HEMA) and triethylene glycol dimethacrylate (TEGDMA) [31]. Increased oxidative stress and the perturbation of intracellular redox homeostasis are supposed to be results of reactive oxygen species (ROS) formed by these monomers [32]. As a consequence, inflammation, inhibition of cell proliferation and apoptosis may result from the release of these monomers [33]. HEMA and TEGDMA are proven to promote inflammatory responses in gingival fibroblasts and the release of prostaglandin E_2_ [34,35], could cause allergic reactions [36] and may inhibit the cell proliferation or differentiation of human dental pulp cells into dentin [37]. Low molecular weight monomers such as HEMA and TEGDMA may diffuse through dentinal tubules, reach the pulp tissue and cause the above-mentioned reactions [38]. Since all these materials are placed in close proximity to the pulp and may exhibit some grade of toxicity due to the release of chemical species, the aim of this study was to identify possible organic eluates released from four intermediate bases by the use of gas chromatography-mass spectrometry (GC-MS), as well as to study the surface microstructure in combination with the elemental composition of these materials with scanning electron microscopy (SEM). To the best of our knowledge, there is no literature data on the investigation of the potential chemical activity of such materials, and the findings of the present work intend to clarify the hypothesis that the studied materials may be associated with possible toxic components.

## 2. Results

**Figure 1:**
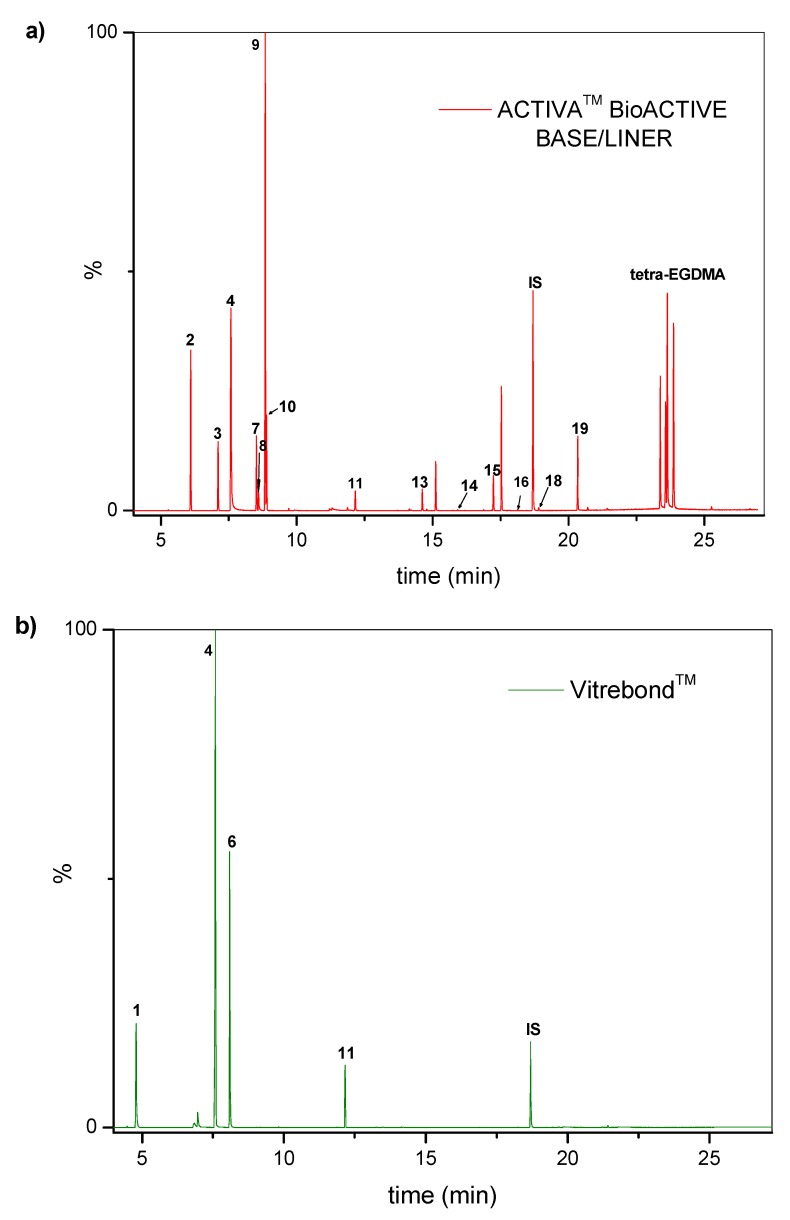

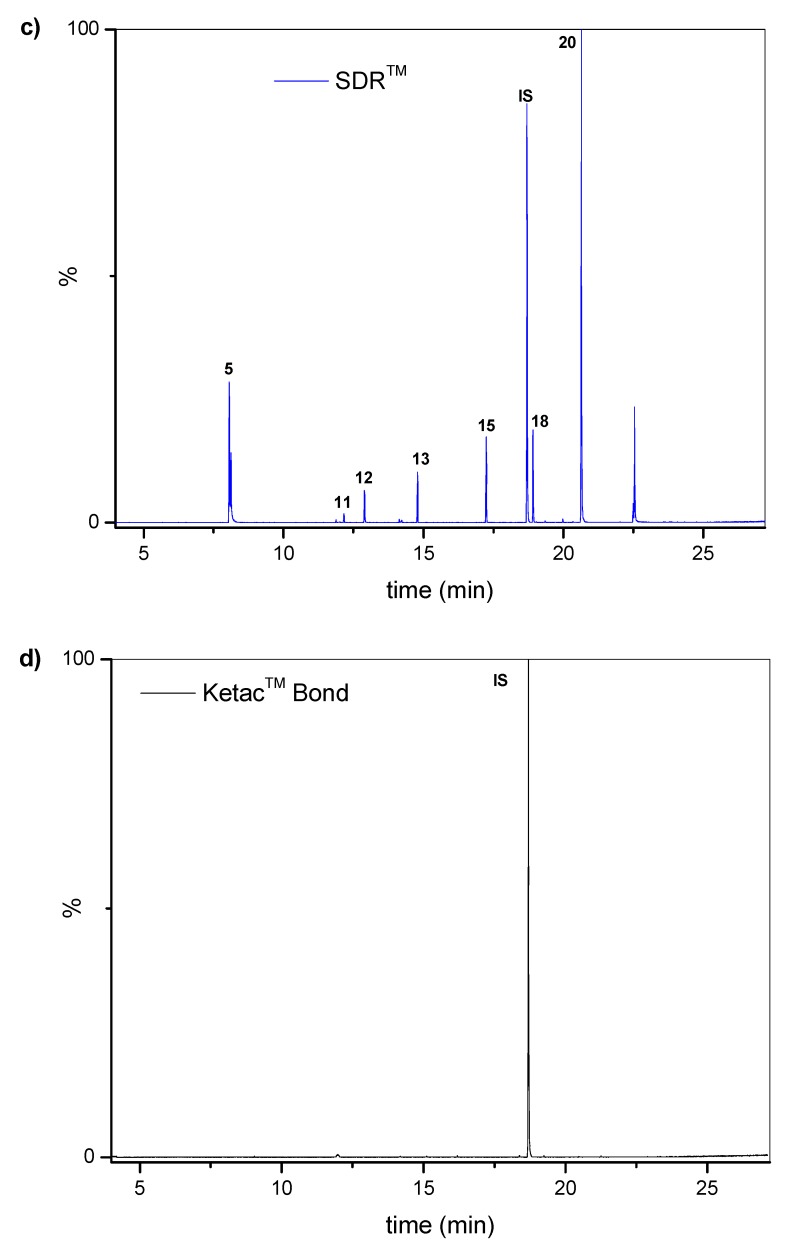
Chromatogram profiles recorded for the methanolic extracts of: (**a**) ACTIVA BioACTIVE BASE/LINER^TM^; (**b**) Vitrebond^TM^; (**c**) SDR^TM^ and (**d**) Ketac^TM^ Bond.

Table 1 accumulates the GC-MS identification data for the total compounds eluted from the examined dental materials. The analytes detected in the methanolic eluent for the specific intermediate base material are summarized in Table 2. Representative GC-MS chromatograms of methanol extracts from each material are depicted in Figure 1, while the mean values and standard deviations of the eluted substances are listed in Table 3. It is obvious that no substances were released from Ketac™ Bond, since this material constitutes a conventional glass ionomer cement and was considered as the control group. However, HEMA and camphorquinone (CQ) were released from both Vitrebond™ and ACTIVA™ BioACTIVE-BASE/LINER™, with Vitrebond™ presenting a significantly higher release of both HEMA and CQ than ACTIVA™ BioACTIVE-BASE/LINER™ (*p* < 0.05). Furthermore, butylated hydroxytoluene (BHT), ethyl 4-(dimethylamino) benzoate (DMABEE) and TEGDMA were released from both ACTIVA™ BioACTIVE-BASE/LINER™ and SDR™. Although the DMABEE release was similar among the two materials, the BHT and TEGDMA elution was significantly higher in SDR™ (*p* < 0.05). Moreover, methoxyphenyl acetic acid (MOPA) was detected only for ACTIVA™ BioACTIVE-BASE/LINER™, while the benzene chloride (BC) and benzene iodide (BI) compounds were unique for the Vitrebond™ organic eluent.

**Figure 2:**
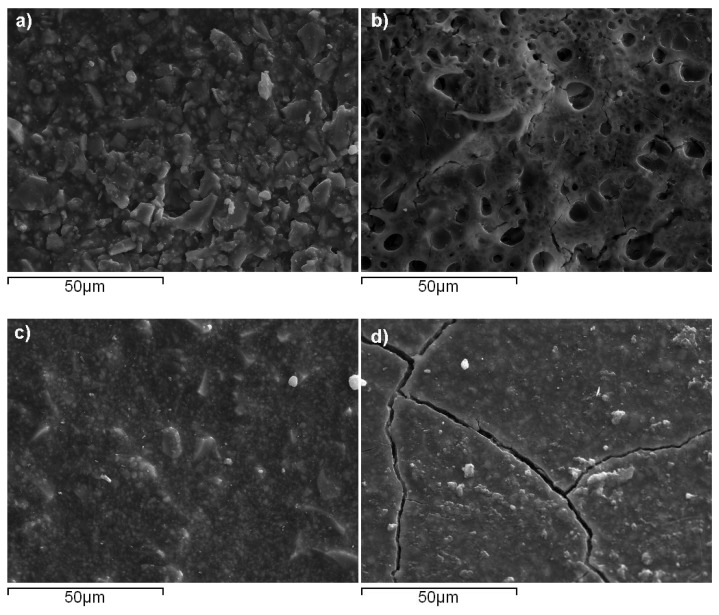
SEM microphotos taken for: (**a**) ACTIVA^TM^BioACTIVE-BASE/LINER; (**b**) Vitrebond^TM^; (**c**) SDR^TM^ and (**d**) Ketac^TM^ Bond specimens (1000× magnification).

**Figure 3:**
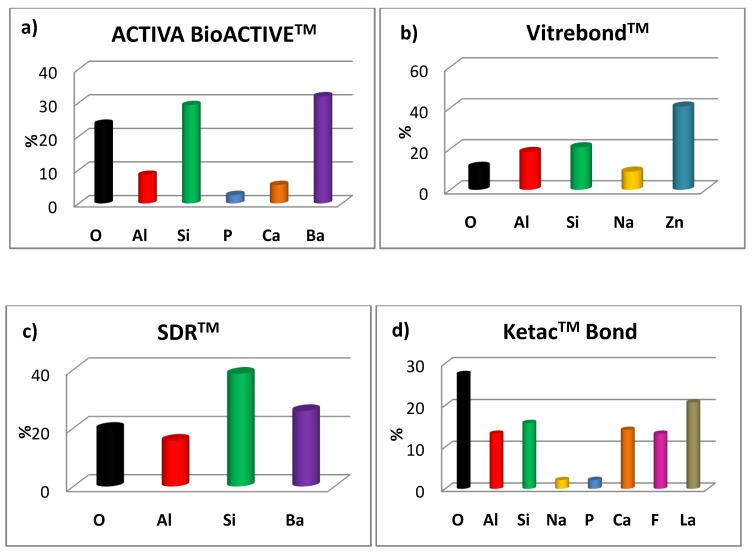
SEM-EDX elemental analysis for: (**a**) ACTIVA^TM^BioACTIVE-BASE/LINER^TM^; (**b**) Vitrebond^TM^; (**c**) SDR^TM^ and (**d**) Ketac^TM^ Bond intermediate bases.

Τhe surface morphological characteristics of the four studied materials captured by SEM is illustrated in Figure 2. It can be seen that Vitrebond^TM^ exhibited small pits approximating a size of up to 10 μm, while Ketac™ Bond presented elongated cracks along with some filler aggregates. On the contrary, no voids were visible for ACTIVA™ BioACTIVE-BASE/LINER™ and SDR™, yielding structural characteristics close to a typical homogeneous surface. The SEM-EDX elemental analysis data for all materials are plotted in Figure 3. Considerable amounts of fluoride (13.2 wt-%) and calcium (14.1 wt-%) were found for Ketac™ Bond (Figure 3d), whereas the phosphorous percentages where almost similar for both ACTIVA™ BioACTIVE-BASE/LINER™ (2.5 wt-%) (Figure 3a) and Ketac™ Bond (2.1 wt-%). Figure 3b reveals the great presence of zinc (40.7 wt-%) found in Vitrebond^TM^. Furthermore, significant quantities of barium were detected in ACTIVA™ BioACTIVE-BASE/LINER™ (31.9 wt-%) and SDR™ (25.9 wt-%) (Figure 3c), while lanthanum (20.69 wt-%) was found on the surface of Ketac™ Bond as well.

## 3. Discussion

According to manufacturer’s MSDS declaration, ACTIVA^TM^ BioACTIVE-BASE/LINER^TM^ is a bioactive ionic resin with reactive glass filler. In particular, it is a blend of diurethane and other methacrylates with modified polyacrylic acid containing amorphous silica and sodium fluoride (Table 4). It is also claimed that ACTIVA^TM^ contains no Bis-GMA, BPA and relative derivatives. In the present study, no BPA could be identified in the chromatographic profile of the aforementioned material (Figure 3a). MOPA was found to be the dominant compound among the other eluted components of the ACTIVA^TM^ extract. Previous studies have proven the fungicidal activities of MOPA [39] and its corresponding derivatives [40] against specific fungal strains. Acetophenone (ACP), and mostly its derivatives like 2,2-dimethoxy-2-phenyl acetophenone (DMPA), are widely spread as type I photoinitiators yielding free-radicals due to a unimolecular bond cleavage [41,42,43,44,45]. DMPA is found to induce cell viability [46]. α-Methylstyrene (MS) and cumene (CM) traces could be possibly associated with α-methylstyrene dimer which in turn has served as a reversible addition-fragmentation chain-transfer (RAFT) agent in the synthesis of branched nanogels, containing UDMA crosslinkers, potentially used as shrinkage and stress-limiting resin additives [47]. However, the high temperature conditions during the gas chromatography analysis process can result in the formation of ACP, MS and methyl cumyl ether (MCE) as potential thermal degradation products of cumyl hydroperoxide or dicumyl peroxide [48,49]. The latter residual constituents in the polymer matrix may be used in redox initiator systems promoting the free radical polymerizations of methacrylate monomers at low temperatures [50]. Furthermore, the DMABEE found in ACTIVA’s organic eluent mixture is a well-known tertiary amine contributing to such a redox initiation system [51]. DMABEE demonstrates a moderate cytotoxic effect, and due to its lipophilic nature it may accumulate in cell membranes and disrupt their integrity [52]. After 24 hours storage in methanol, the lower eluted fraction of the CQ initiator, compared to the DMABEE and 2-(dimethylamino)ethyl methacrylate (DMAEMA) co-initiators, may be attributed to the steric bulk of the chiral structure of CQ, which could be responsible for lower diffusion rates to methanol despite the relatively low molecular weight (196.22 g/mol). HEMA was found to be the secondabundant eluate, even if it is not declared in the corresponding SDS. Provided that ACTIVA^TM^ is considered a resin-modified glass ionomer, HEMA may finally react with the polyacid and thus contribute to the formation of the resin-modified polyacid chain, as well as act as a potential co-monomer in the crosslinking process [53]. Moreover, HEMA has been correlated with UDMA thermal fragmentation in the GC injector unit [29] and could be an indirect evidence of the unreacted UDMA monomer [1]. HEMA, due to its low molecular weight and high hydrophilicity, may diffuse through dentin, reach the pulp and cause adverse pulpal reactions [54]. In addition, the detected (trimethylolpropane trimethacrylate) TMPTMA is a tri-functional methacrylate monomer usually used in dental composite resins’ formulations to prompt improved flexural strength, hardness, absorption, and crosslink density [55] and wear properties [56] by acting as a crosslinking agent in the polymer matrix. The chromatogram pattern in the elution range of 23.37–23.86 min has been also shown by other researchers for different capping materials, corresponding to high molecular weight methacrylates (tetra-EGDMA) [1]. 

Regarding the Vitrebond^TM^, GC-MS analysis revealed that HEMA was the most leachable organic component after 24 h aging in methanol. Indeed, this finding fits the MSDS information (Table 4) where HEMA is mentioned in the glass-ionomer liquid composition. BC and BI seem, rather, to be degradation products of diphenyliodonium chloride (DPICl), which has been previously reported as an accelerator of 3-component photoinitiator systems [57,58]. Iodonium salts are capable of photosensitizer’s regeneration by inserting active phenyl initiating radicals, and by substituting inactive terminating radicals and concurrently adding new active phenyl radicals [59,60]. Furthermore, the presence of DPICl in the organic leachate is in line with the SDS statement. 

SDR™ is a bulk-fill restorative material consisting of the base monomers UDMA and ethoxylated bisphenol-A dimethacrylate (Bis-EMA) and the co-monomer TEGDMA, according to the respective MSDS (Table 4). No BPA or other relative derivatives were detected in the methanol leachate after 24 h conditioning. The organic eluent mixture was found to be enriched with oxybenzone (HMBP), which is a UV stabilizer frequently incorporated in cosmetics, personal care products, coating products, fillers, putties, plasters, modelling clay and finger paints [52]. 2-Hydroxypropyl methacrylate (HPMA) and TEGDMA were also eluted, revealing the existence of two methacrylate co-monomers in the SDR™ formulation, while HPMA played a dualrole similar to HEMA, as described above. Additives like BHT, identified in the SDR™ chromatogram, ensure the stability during storage by polymerization inhibition through consuming free radicals that are formed spontaneously [61]. The presence of the DMABEE accelerator as an eluent, accompanied by a CQ weak detection, denotes either a complete degradation of the main initiator to generate free radicals or possible obstacles dealing with diffusion phenomena. 1,6-hexamethylene diisocyanate (HMDI) is a representative starting material used in the synthesis of urethane dimethacrylate monomers [62], which are contained in the SDR™ formulation. 

In contrast to the aforementioned materials, Ketac^TM^ Bond did not provide an organic-enriched mixture in methanol, as was expected, because it is a typical glass ionomer cement (Table 4), and hence the setting reaction does not involve any organic monomers and other initiating additives. Furthermore, no traces of tartaric acid were found, indicating a full consumption during the setting process.

Regarding the SEM-EDX analysis, the occurrence of oxygen, aluminum, silicon, phosphorous and calcium in all materials could be generally attributed to the oxides of aluminosilicate glass fillers used in glass ionomer cements [63]. In particular, the obtained results for Ketac^TM^ Bond denoted that a relatively brittle surface due to the absence of resin, as shown in Figure 2d, may favor the formation of partial higher surface areas enriched with high fractions of elements like fluoride and calcium available to be analyzed. On the other hand, the rest of the investigated intermediatebases contain resin, which probably prevents the accumulation of elements on the surface layers, while the penetration of some elements in the ionic form from the bulk to the surface might be activated through the chemical interactions in aqueous media. Indeed, Akbulut et al. conducted an SEM-EDX for SDR™ and Vitrebond^TM^ after incubation with periodontal ligament fibroblasts (PDL) cell media, and the results showed that a higher number of elements remained on specimens’ surfaces, including a high ratio of fluoride in the case of Vitrebond^TM^ [64]. Nevertheless, the small amount of detected phosphorous for ACTIVA^TM^ BioACTIVE-BASE/LINER^TM^ confirms the manufacturer’s claim about a water-friendly ionic resin containing phosphate acid functionality with antibacterial activity, whereas resin-glass fillers and intermediate base-tooth structure interactions are also sustained in this way. As result, a robust resin-hydroxyapatite complex can be formed through a phosphate group hydrogen ions replacement by calcium in the tooth structure. Barium found on ACTIVA^TM^ and SDR™ surfaces is usually associated with the existence of the radiopacifiying filler agent BaO [65]. In terms of Vitrebond^TM^, the identified zinc has also been reported by other researchers [64], and its bacterial properties have also been studied comprehensively [66]. The large content of lanthanum for Ketac^TM^ Bond can be attributed to its frequent incorporation in the powder constituents of glass-ionomer cements for an opaqueness increase against rays [67] and was also measured by other researchers [68].

## 4. Materials and Methods 

### 4.1. Specimens’ Preparation

Four commercially available intermediaterestoratives were used for this study: ACTIVA™ BioACTIVE-BASE/LINER™ (PULPDENT™ Corporation, Watertown, Massachusetts, USA), Ketac™ Bond Glass Ionomer (3M™ ESPE™, St. Paul, Minnesota, USA), SDR™ (DENTSPLY DeTrey GmbH, Konstanz, Germany) andVitrebond™ Light Cure Glass Ionomer Liner/Base (3M™ ESPE™, St. Paul, Minnesota, USA). Detailed information about their composition, according to manufacturers, is shown in Table 4. 

Five specimens of each material were prepared according to the following procedure:100 mg of uncured material was used to fill Teflon molds in order to produce disks (6 mm diameter, 1 mm thickness). The polymerization of the disks was performed using a curing LED light (Bluephase style, Ivoclar/Vivadent, Amherst, New York, USA, power ranged 1100–1400 mW/cm^2^), which was applied for 20 s directly on the surface of the samples. Three identical repetitions ofthe experiment were conducted.

### 4.2. Elution Evaluation

A solution of 1ml methanol (Methanol, HPLC gradient grade 99.9+%, CHEM-LAB, Zedelgem, Belgium) containing 0.1 mg/ml caffeine (Caffeine 99%, Alfa Aesar, Kandel, Germany) as internal standard was placed in separate glass tubes, and each sample was immersed in the solution.The glass tubes were secured with a ground glass stopper to prevent evaporation. After 24 hours, the solutions were transferred to separate GC vials and injected into the gas chromatograph.

### 4.3. Separation by Gas Chromatography and Mass Spectrometric Detection

The analyses were performed by using a gas chromatography-mass spectrometry instrument (GC-MS Clarus 500, Perkin Elmer, Shelton, Connecticut, USA) supported by a suitable software (Perkin Elmer, TurboMass version 5.4.2). The GC unit was equipped with an autosampler and a DB-5-MS capillary column (30 m, 0.25 mm id., 0.25 μm film, Agilent, Santa Clara, California, USA). The injector (split 1:20) was held at 250 °C. After a constant temperature of 50 °C for 2min, the oven’s temperature increased until 300 °C and remained constant for 5 min. As a carrier, gas Helium 5.0 was used with a constant flow rate of 1 mL/min. The transfer line from GC to MS was set to 310 °C. The mass spectrometer was operated in electron ionization mode (E.I.), while the ion source was operated at 220 °C. Only positive ions were scanned. The syringe was rinsed two times before and after injection.

The identification and quantification of the analytes were performed by using a mass spectrometer in full scan mode scanning from 50 to 450 *m/z* at a rate of 0.2 scans per second.NIST library (National Institute of Science and Technology, Gaithersburg, MD, USA), retention time and literature data were used for the identification of different compounds. The internal standard, caffeine, was analyzed at the m/z ratio of 194 and was used for quantification, and each unknown peak was normalized to the caffeine peak. Reagent blank samples, only containing caffeine dissolved in methanol, were also analyzed. In order to prevent carry-over effects, methanol was injected between all samples. 

### 4.4. Scanning Electron Microscopy Characterization (SEM)

Two specimens of each material (5 × 5 × 5 mm) were prepared and stored at 37 °C. Then, the samples were mounted in resin and were ground using discs and pastes with an automatic polishing machine. Prior to mounting on aluminum stubs, they were carbon-coated to avoid charging under the electron beam. They were viewed under the scanning electron microscope (JEOL, JSM-6390LV, JEOL USA, Inc., Peabody, Massachusetts, US) equipped with an energy dispersive X-ray (EDX) microanalytical system (INCA PentaFETx3, Oxford Instruments, Abingdon, England). Scanning electron micrographs of the different material microstructural components at different magnifications in back-scatter electron mode were captured. The elemental analysis of the specimens’ surfaces was carried out by an Energy Dispersive X-ray microanalysis.

### 4.5. Statistical Analysis

The results are presented as mean values with associated standard deviations. A statistical analysis was performed for eluted substances from at least two materials using IBM SPSS software, version 25 with an assumed level of significance *p* < 0.05. 

## 5. Conclusions

Different organic substances were eluted from each material, and the substances’ elution was dependent on the material’s composition. Moreover, the resin matrix of some intermediate dental materials may inhibit the elements’ accumulation on the surface layers. The findings, based on the leachate’s chemical analysis, confirmed the initial hypothesis that the examineddental materials could be associated with chemical species that may potentially exhibittoxicity.

## Figures and Tables

**Table 1:** Intermediate restoratives’ elutedsubstances arranged by increasing retention time, with the abbreviation, molecular formula, compound name, molecular weight, characteristic ionsand chemical structure.

Eluate	Retention Time (RT)	Abbreviation	Molecular Formula	Compound Name	Molecular Weight	Characteristic Ions, *m/z*	Chemical Structure
1	4.79	BC	C_6_H_5_Cl	Benzene chloride	112	112, 77, 114, 51	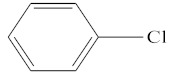
2	6.08	CM	C_9_H_12_	Cumene	120	105, 120, 77	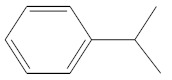
3	7.08	MS	C_9_H_10_	α-Methylstyrene	118	118, 103, 78, 115	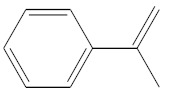
4	7.55	HEMA	C_6_H_10_O_3_	2-Hydroxyethyl methacrylate	130	69, 87	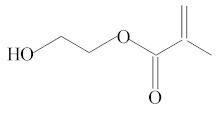
5	8.06	HPMA	C_7_H_12_O_3_	2-Hydroxypropyl methacrylate	144	69, 100, 99, 58	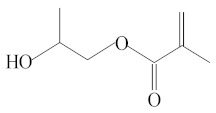
6	8.09	BI	C6H5I	Benzene iodide	204	77, 204	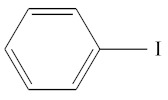
7	8.51	ACP	C_8_H_8_O	Acetophenone	120	105, 77, 120, 51	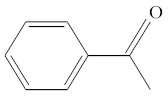
8	8.58	DMAEMA	C_8_H_15_NO_2_	2-(Dimethylamino)ethyl methacrylate	157	58, 71	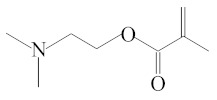
9	8.82	MOPA	C9H10O3	Methoxyphenyl acetic acid	166	121, 77, 51, 78	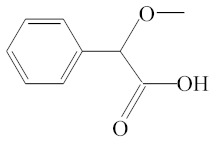
10	8.87	MCE	C_10_H_14_O	Methyl cumyl ether	150	135, 91, 77, 73, 136	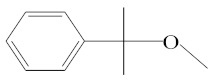
11	12.14	CQ	C_10_H_14_O_2_	Camphorquinone	166	95, 69, 83	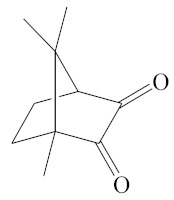
12	12.89	HMDI	C_8_H_12_N_2_O_2_	1,6-Hexamethylene diisocyanate	168	56, 85, 69	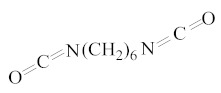
13	14.77	BHT	C_15_H_24_O	Butylated hydroxytoluene	220	205, 220, 57	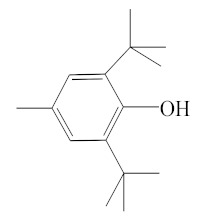
14	15.91	DEGDMA	C_12_H_18_O_5_	Diethyleneglycoldimethacrylate	242	69, 113	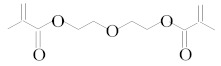
15	17.23	DMABEE	C_11_H_15_O_2_N	Ethyl 4-(dimethylamino)benzoate	193	148, 193, 164	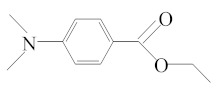
16	18.13	BDBTF	C_13_H_15_F_3_	Benzene, 1-(1,3-Dimethyl-2-Butenyl)4-(trifluoromethyl)-	228	69, 87	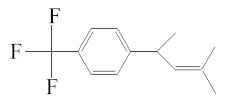
17	18.69	IS	C_8_H_10_O_2_N_4_	Caffeine	194	194, 109	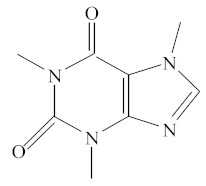
18	18.91	TEGDMA	C_12_H_18_O_5_	Triethylene glycol dimethacrylate	286	69, 113	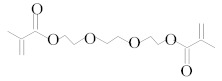
19	20.34	TMPTMA	C_18_H_26_O_6_	Trimethylolpropane trimethacrylate	338	69, 253	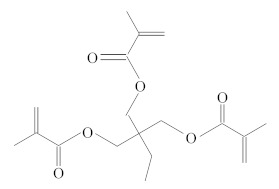
20	20.64	HMBP	C_14_H_12_O_3_	Oxybenzone	228	227, 151, 228, 77	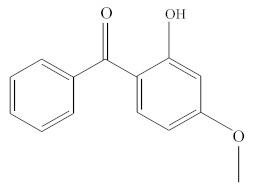

**Table 2:** Analytes detected in the methanol leachate of the four investigated materials. The numbers correspond to the eluates reported in Table 1.

	Analyte																		
Material	1	2	3	4	5	6	7	8	9	10	11	12	13	14	15	16	18	19	20
ACTIVA^TM^ BioACTIVE- BASE/LINER^TM^		√	√	√			√	√	√	√	√		√	√	√	√	√	√	
Vitrebond^TM^	√			√		√					√								
SDR^TM^					√						√	√	√		√		√		√
Ketac^TM^ Bond (3M ESPE)																			

**Table 3:** Median values (Interquartile range) ofrelative amounts (%CF) for eluates measured (*n* = 15) in methanolic extracts after 24 hours conditioning at 37 °C.

Intermediate Base	Eluate	%CF 1 Day
ACTIVA^TM^ BioACTIVE-BASE/LINER^TM^	ACP MS BDBTF BHT CQ CM DEGDMA DMABEE	28.59 (6.76) 24.35 (3.94) 0.46 (0.03) 0.49 (0.12) 7.43 (1.18) 54.57 (16.89) 0.24 (0.10) 13.34 (1.66)
	DMAEMA HEMA MOPA MCE TEGDMA TMPTMA	7.72 (1.14) 123.71 (22.37) 170.29 (37.22) 30.94 (7.14) 2.13 (2.92) 29.32 (3.39)
Vitrebond^TM^	BC BI	140.41 (64.76) 322.77(119.29)
	CQ HEMA	49.69* (23.84) 665.65* (414.47)
SDR^TM^	BHT CQ DMABEE HMBP HMDI HPMA	7.43** (0.83) 2.35 (0.66) 12.35 (2.85) 102.68 (6.02) 4.60 (1.24) 20.27 (5.69)
	TEGDMA	14.69** (3.02)
Ketac^TM^ Bond	-	-

* indicates statistical significance among elutes from VitrebondTM&ActivaTMBioACTIVE-BASE/LINERTM, ** indicates statistical significance among eluates from SDRTM&ActivaTMBioACTIVE-BASE/LINERTM.

**Table 4:** Specifications of dental materials used in the study, according to the data provided by the manufacturers.

Material	Company	MSDS Synthesis	wt-%	LOT No
ACTIVA™ BioACTIVE-BASE/LINER™	PULPDENT™ Corporation, Watertown, Massachusetts, USA	Blend of diurethane and other methacrylates with modified polyacrylic acid	53.20%	170731
		Silica, amorphous	3%	
		Sodiumfluoride	0.90%	
Ketac™ Bond GlassIonomer	3M™ ESPE™, St. Paul, Minnesota, USA	Water	80%–90%	3511154
		Tartaricacid	10%–20%	
SDR^TM^	DENTSPLY DeTrey GmbH, Konstanz, Germany	Urethane dimethacrylate resin	10%<25%	1612000538
		Ethoxy bisphenol-A dimethacrylate	2.5%<10%	
		2,2′-Ethylenedioxydiethyldimethacrylate (Triethylene glycol dimethacrylate)	2.5%<10%	
Vitrebond™ Light Cure Glass Ionomer Liner/Base	3M™ ESPE™, Minnesota, St. Paul, USA	Powder:		Ν873542
		Glasspowder	>95%	
		Diphenyliodonium chloride	<2%	
		Liquid:		
		Copolymer of acrylic and itaconic acids	35%–45%	
		Water	30%–40%	
		2-hydroxyethyl methacrylate	20%–30%	
